# Functional heterogeneity of MCT1 and MCT4 in metabolic reprogramming affects osteosarcoma growth and metastasis

**DOI:** 10.1186/s13018-023-03623-w

**Published:** 2023-02-22

**Authors:** Gaohong Sheng, Yuan Gao, Hua Wu, Yang Liu, Yong Yang

**Affiliations:** 1 Department of Orthopedics, Tongji Hospital, Tongji Medical College, Huazhong University of Science and Technology, Jiefang Avenue 1095, Wuhan, 430030 China; 2 Department of Oncology, Tongji Hospital, Tongji Medical College, Huazhong University of Science and Technology, Wuhan, 430030 China

**Keywords:** MCT1, MCT4, Metabolism, Oxidative stress, Osteosarcoma

## Abstract

**Background:**

Osteosarcoma is the most common primary malignant bone tumor in adolescents and children and prone to develop lung metastasis. Its prognosis has been virtually unimproved over the last few decades, especially in patients with metastases, who suffer from a dismal survival. Recently, increasing attention has been devoted to monocarboxylate transporters-related (MCTs) metabolic reprogramming. However, the role of MCT1 and MCT4 in osteosarcoma progression and the underlying mechanisms remain to be further elucidated.

**Methods:**

In this study, we established MCT1 and/or MCT4 knockout cell lines by CRISPR/Cas9 genome editing technology. Then, we assessed glycolysis and oxidative phosphorylation capacities by measuring lactate flux and oxygen consumption. We also performed flowcytometry to test circulating tumor cells and PET/CT to evaluate glucose uptake.

**Results:**

MCT1 was found to be involved in both glycolysis and oxidative respiration due to its ability to transport lactate in both directions. MCT1 inhibition significantly reduced circulating tumor cells and distant metastases partially by increasing oxidative stress. MCT4 was primarily related to glycolysis and responsible for lactate export when the concentration of extracellular lactate was high. MCT4 inhibition dramatically suppressed cell proliferation in vitro and impaired tumor growth with reduction of glucose uptake in vivo.

**Conclusions:**

Our results demonstrate the functional heterogeneity and redundancy of MCT1 and MCT4 in glucose metabolism and tumor progression in osteosarcoma. Thus, combined inhibition of MCT1 and MCT4 may be a promising therapeutic strategy for treating tumors expressing both transporters.

**Supplementary Information:**

The online version contains supplementary material available at 10.1186/s13018-023-03623-w.

## Background

Despite being a rare cancer, osteosarcoma (OS) is the most common primary malignant bone tumor in adolescents and children, with an incidence of approximately 1–3/million/year worldwide [1]. OS tends to occur in the metaphysis of long bones, most commonly in the distal femur, proximal tibia and humerus [2]. A distinctive feature of OS is to develop metastases throughout the course of disease, with lung being the most frequent site of metastasis followed by bone as the second. Since the introduction of chemotherapy as an adjunct to surgery in the early 1970s [3], the five-year survival rate has improved significantly to around 70% in localized disease, however, there has been limited improvement in the last few decades. More importantly, the long-term survival of patients with metastases or relapse remains virtually unchanged at only about 20% [4]. Therefore, novel treatments are urgently needed to break this clinical dilemma. Collaborative efforts including biological, preclinical and clinical strategies are essential to increase understanding of OS biology, explore molecular aberrations and thus identify potentially promising targets.

The solute carrier family 16 (SLC16), also known as monocarboxylate transporters (MCTs), is comprised of 14 members that play a critical role in the transport of short-chain monocarboxylates, amino acids, hormones and nutrients [5, 6]. Due to the wide range of substrates, MCTs are involved in a variety of biologic pathways, including energy metabolism, T cell activation, thyroid hormone metabolism, pancreatic B cell dysfunction and drug delivery [7]. Among these 14 isoforms, MCTs 1–4 are well characterized as proton-linked monocarboxylate transporters while the other MCT members are less investigated. MCTs 1–4 are associated with the transfer of metabolites, such as lactate, pyruvate, and ketone bodies (i.e., acetoacetate and β-hydroxybutyrate) across plasma membrane [8].

Cancer cells are highly metabolically plastic entities that adapt to different stimuli (i.e., nutrients, O_2_ concentration, biological activities and genetic mutations) and thus exhibit different metabolic phenotypes. Recently, lactate has been considered not only as a major fuel for oxidative tumor cells, but also as a pivotal signaling molecule to regulate gene expression. Although lactic acid is not the only substrate for MCTs, it is the most abundant and most widely studied in human [9]. The lactate level in resting healthy tissue is around 1 mM, while that in tumor tissue can be elevated up to 10–40 mM [10, 11]. Notably, increasing attention has been attracted to the role of lactate metabolism in tumor development and progression. Faubert et al. has confirmed that tumor cells autonomously take up lactate for tricarboxylic acid (TCA) cycle through MCT1 in vivo, which is more evident in aggressive tumors [12]. The overexpression of MCT1 and MCT4 has been documented in various cancers and is associated with poor prognosis of patients [9]. Considering functional similarities and synergistic effects between MCT1 and MCT4, simultaneous inhibition of both transporters appears to be more effective in suppressing tumors [13, 14]. Although MCT1 inhibition by α-Cyano-4-hydroxycinnamate (CHC) has been found to reduce OS malignancy, CHC is not specific for MCT1 [15]. Furthermore, whether there are synergistic effects and functional differences between MCT1 and MCT4 remains unknown.

Therefore, the aim of our article is to explore the role of MCT1 and MCT4 in OS metabolic remodeling, their impact on tumor growth and metastasis and the potential mechanisms through in vivo and in vitro experiments. MCTs is closely related to glucose metabolism and in particular MCT1 and MCT4 have been identified as lactate transporters and intensively investigated in cancers. We firstly examined the function of MCT1 and MCT4 in lactate transport in OS cells by pharmacological inhibition and gene knockout. Then we explored the relationship between MCT1/4 and OS biological behaviors, such as proliferation and invasiveness. Moreover, a recent article reported that MCT1-mediated metabolic heterogeneity confers differences in metastatic potential [16]. As we know, OS has a high tendency to develop distant metastases and leads to a poor prognosis. Thus, our focus is on the functional heterogeneity of MCT1 and MCT4 in glucose metabolism and OS metastasis. We aim to discover the underlying mechanisms by which MCT1 and MCT4 affect OS growth and metastasis by metabolic remodeling. Besides, the interaction between MCT1 and MCT4 was also studied. The research on cancer metabolism can increase our understanding of OS biology and discover potential targets, especially the metabolic features associated with metastasis. By developing novel therapeutic strategies that target metabolic key nodes, it is promising to further improve the survival of OS patients, even with metastasis.

## Methods

### Cell culture and treatment

MNNG/HOS and U-2 OS cell lines were kindly provided from the Cell Bank of Chinese Academy of Sciences (Shanghai, China). In general, cells were cultured in Dulbecco’s modified Eagle’s medium F12 (DMEM/F12; Gibco, USA) supplemented with 10% fetal bovine serum (FBS; Gibco, USA) and 1% penicillin/streptomycin (Sigma-Aldrich, USA). Cells were maintained at 37 °C in normoxic condition with 5% CO_2_ in a humid incubator (Thermo Fisher Scientific, USA). In the case of hypoxia, cells were incubated under 1% O_2_. When cultured cells grow up to about 80% confluence, they were digested for subsequent experiments. The reagents AZD3965 (10 μM), VB124 (10 μM), oligomycin (1 μM), and N-acetyl-cysteine (NAC, 200 μM) were purchased from Selleck and used for designated time.

### Construction of cell lines

MCT1 (SLC16A1) or MCT4 (SLC16A4) knockout (KO) cell lines were constructed by CRISPR/Cas9 genome editing technology. The single guide RNA (sgRNA) was cloned into pLentiCRISPR v2 (Plasmid #52961, Addgene) and then the constructed plasmids were transfected into 293T cells to generate lentivirus. Cells were infected by synthesized lentivirus and screened by puromycin. Cells with puromycin resistance were seeded into a 96-well plate at a monoclonal density and then clones were expanded. The protein expressions were detected by western blot to screen clones for MCT1 or MCT4 deletion. For MCT1/4 double KO cell lines, MCT1 KO cells were infected again with the lentivirus containing sgRNA targeting MCT4. Clones were cultured for screening by protein expression assays. The targeted sequences for human MCT1 are sgRNA #1 5′–ACGTGACTGGCTAGCTGCGT–3′ and sgRNA #2 5′–ACAGACGTATAGTTGCTGTACGG–3′, and for human MCT4 are sgRNA #1 5′–GAAGAGGCATCATGCTGAAG–3′ and sgRNA #2 5′–TAATCTGACTGACCGTCTCAAGG–3′, respectively.

### Lactate and ATP measurement

For lactate level assay, 1 × 10^5^ cells were seeded into a 12-well plate and grew overnight. The medium was replaced by FBS-free DMEM/F12 followed by incubation for 2 h. Then, the supernatant was collected for specific reactions according to the protocol of Lactate Assay Kit II (Biovision, USA). The reaction mixture was protected from light at room temperature for 30 min. Lactate level was measured at 450 nm by a microplate reader (Bio-Rad, USA). Additionally, we also detected the lactate level within tumor tissue.

ATP Colorimetric Assay Kit (Biovision, USA) was used to analyze ATP production. According to the manufacturer’s protocol, a total of 5 × 10^5^ cells were collected and extracted with ATP Assay Buffer. After centrifugation at 1.2 × 10^4^ rpm for 5 min, the supernatants were collected for further reaction for 30 min in the dark at room temperature. Optical density (OD) values of reaction mixture were measured at 570 nm using a microplate reader. All above measurements were normalized to protein or tumor tissue weight.

### Oxygen consumption rate assay

Cellular oxygen consumption rate (OCR) was measured by Seahorse XFe 24 Extracellular Flux Analyzer (Seahorse Bioscience, USA) with Seahorse XF Cell Mito Stress Test Kit (Agilent Technologies, USA), which represents mitochondrial respiration capacity. According to the manufacturer’s instructions, 2 × 10^5^ cells per well were seeded into a Seahorse XF 24 microplate. When cells were just spread over the plate wells, they were prepared for OCR detection. During the measurement of OCR, 1.5 μM oligomycin, 1 μM Carbonyl cyanide-4 (trifluoromethoxy) phenylhydrazone (FCCP) and 0.5 μM rotenone plus antimycin A (Rote/AA) were sequentially injected into each well at designated time points. Values were normalized to protein concentration.

### Western blot analysis

Cells were lysed by using radio immunoprecipitation assay lysis buffer (RIPA) supplemented with 1% protease and phosphatase inhibitors (Boster, Wuhan, China) and protein concentration was measured with Bicinchoninic Acid Assay (BCA) protein assay kit (Boster, Wuhan, China). Equal amounts of protein samples (15–25 μg) were added into each lane and separated on 4–20% SDS-PAGE gels. Then, proteins in the gel were transferred to polyvinylidene difluoride (PVDF) membranes (Millipore, USA). Membranes were then blocked in 5% bovine serum albumin (BSA) or 5% milk in Tris-buffered Saline (TBS) containing 0.1% Tween-20 (TBST) for 1 h at room temperature. After blocking, membranes were incubated in specific primary antibodies at 4 °C overnight. On the next day, membranes were rinsed with TBST with TBST and subsequently incubated with horse-radish peroxidase (HRP)-conjugated secondary antibodies (Sigma-Aldrich, USA) for 1 h at room temperature. After washing, signals were developed by using an enhanced chemiluminescence (ECL) System (Thermo Fisher Scientific, USA). The antibodies used in this study and their dilutions were as follows, including anti-MCT1 (20139-1-AP, Proteintech; 1:1000), anti-MCT4 (22787-1-AP, Proteintech; 1:1000), anti-HK2 (#2106, Cell Signaling Technology; 1:1000), anti-LDHA (#3582, Cell Signaling Technology; 1:1000), anti-LDHB (ab53292, Abcam; 1:5000), anti-CS (ab96600, Abcam; 1:1000), anti-HIF-1α (#14179, Cell Signaling Technology; 1:1000), anti-c-Myc (ab32072, Abcam; 1:1000), anti-p65 (#9936, Cell Signaling Technology; 1:1000), anti-PCNA (#2586, Cell Signaling Technology; 1:1000), anti-CDK4 (#12790, Cell Signaling Technology; 1:1000), anti-MMP2 (#87809, Cell Signaling Technology; 1:1000), anti-MMP9 (#13667, Cell Signaling Technology; 1:1000), and anti-Tubulin (ab52866, Abcam; 1:10000).

### Cell proliferation assay

Cell Counting Kit-8 assay (CCK-8; Boster, China) was used to evaluate cell proliferation after specific treatments. Briefly, culture medium was replaced with 100 μl fresh medium containing 10 μl CCK-8 solution in each well of a 96-well plate. Then, cells were incubated at 37 °C for 2 h and OD value was read at 450 nm by a microplate reader (Bio-Rad, USA).

### Wound healing migration assay

Cells were seeded in a 12-well plate and starved overnight with serum-free medium when cells reached approximately 90% confluence. A 200 μl sterile pipette tip was used to create a scratch to mimic a wound. After washing, fresh medium with 2% FBS was added. At indicated time points, images were captured by a microscope (EVOS FL Auto Imaging System, Life technologies, Gaithersburg, MD) and the percentage of wound healing was calculated by comparison with initial would area.

### Matrigel invasion assay

The invasion assay was performed by using a transwell insert with an 8-μm microporous polycarbonate membrane (Corning, USA). Chamber inserts were precoated with Matrigel (BD Biosciences, USA) and 2 × 10^4^ cells in 100 μl serum free medium were seeded on the upper surface of the insert. Medium containing 10% FBS was added to the lower compartment. After incubation at 37 °C for 24 h, those cells that crossed Matrigel toward the lower surface of the insert were fixed with 4% formalin, followed by 0.1% crystal violet staining. The invaded cells were then observed under a microscope and counted using ImageJ Fiji.

### NAD+/NADH, NADP+/NADPH, GSH/GSSG analyses

NAD+/NADH assay kit, NADP+/NADPH assay kit, and GSH/GSSG assay kit were purchased from Beyotime (Shanghai, China) and used to measure the levels of total NAD (NADt) and NADH, total NADP (NADPt) and NADPH, and total GSH (GSHt) and GSSG according to the manufacturer’s instructions, respectively. Briefly, cells or homogenized tumor tissues were lysed and the supernatants were collected by centrifugation. For NAD+/NADH and NADP+/NADPH assays, NADt (NAD+ and NADH) and NADPt (NADP+ and NADPH) levels were first determined at OD 450 nm. Then, the amounts of NADH and NADPH were measured by decomposing NAD+ and NADP+ at 60 °C for 30 min, respectively. We calculated NAD+ level by the following formula: NAD+  = NADt – NADH, as well as NADP+. For GSH/GSSG analysis, GSHt (GSH and GSSG) level was detected by recording OD values at 412 nm. An appropriate amount of GSH scavenging reagent working solution was added to the supernatant and then the reaction mixture was incubated at 25 °C for 1 h. The isolated GSSG level was then obtained at 412 nm. The GSH level was determined by the following formula: GSH = GSHt – 2 × GSSG. All values were normalized to protein weight of cell lysate or tissue homogenates.

#### Immunofluorescence

The medium was removed and cells were washed 3 times with PBS for 5 min each. Then, cells were fixed in 4% paraformaldehyde for 20 min, followed by permeabilization with 0.5% Triton X-100 for 15 min at room temperature. After blocking with 10% goat serum in PBS for 30 min, cells were incubated with anti-c-Myc (ab32072, Abcam; 1:100) and anti-p65 (#9936, Cell Signaling Technology; 1:200) primary antibodies overnight at 4 °C. The next day, cells were washed for 3 times and then stained with fluorescence labeled secondary antibodies for 1 h in the dark at room temperature, including Alexa Fluor 488- (# A-11008, Invitrogen; 1:500) and Alexa Fluor 647-Goat anti-Rabbit IgG (# A-21244, Invitrogen; 1:500). Cell nuclei were counterstained with DAPI. Images were captured by using a fluorescent microscopy (EVOS FL Auto Imaging System, Life technologies, Gaithersburg) and analyzed by ImageJ Fiji.

#### Mouse xenograft model

Four-week-old female BALB/c nude mice were obtained from the Experimental Animal Center of Huazhong University of Science and Technology (Wuhan, China). Animal experiments were approved by the Ethics Committee of Huazhong Science and Technology University and carried out in accordance with the institutional guidelines for laboratory animal care. Mice were housed in a barrier environment, meeting the requirements for keeping specific pathogen free (SPF) animals. Mice were regularly fed by dedicated staff with sterile water and feed. The breeding environment temperature is around 22 °C with daily temperature difference below 4 °C. The relative humidity is between 50 and 60% and the noise level is under 60 decibels (dB). For in situ tumor models, 50 μl PBS containing 2 × 10^6^ MNNG/HOS cells were injected into the intramedullary cavity of femur by using a 25-gauge needle. Mice were sacrificed 4 weeks after injection and tumors were harvested. We obtained blood samples by cardiac puncture for flow cytometry analysis. Three mice with medium-sized tumors were used to evaluate glucose uptake by PET/CT imaging. The weight of tumor tissue was measured and the number of lung metastases were also counted. A portion of the fresh tissue was immediately prepared for protein and specific biochemical substances assays, including NAD+/NADH, NADP+/NADPH, GSH/GSSG analyses. The remaining tumor tissues were fixed with 4% paraformaldehyde for immunohistochemical stainings. In order to further investigate the frequency of circulating tumor cells, we established an animal model by injecting tumor cells (2 × 10^6^ cells in 100 μl PBS) directly into the tail vein. NAC (100 mg/kg) or saline was given intraperitoneally once daily. Two weeks after treatment, blood acquired by cardiac puncture was used to assess the frequency of circulating tumor cells through flow cytometry.

#### ^18^F-FDG PET/CT

Mice were fasted for 8 h before PET/CT examination. A total of 1.85 MBq (50 μCi) ^18^F-FDG was intraperitoneally injected into each mouse. Then, mice were weighted, anesthetized and fixed on the testing platform. The CT scan was performed for subsequent anatomical localization. One hour after ^18^F-FDG administration, static images were acquired by an animal PET/CT miniEXPLORER (united imaging, China) for 10 min. To obtain higher resolution images, data were reconstructed through a three-dimensional ordered subsets expectation maximization (OSEM) algorithm (4 OSEM iterations, requested resolution: 0.6 mm). Additionally, scatter, attenuation, and decay corrections were performed as well. Maximum standard uptake value (SUVmax) and mean SUV (SUVmean) were measured to estimate glucose uptake in vivo.

#### Immunohistochemistry analysis

Fixed tumor tissues were embedded in paraffin and were sliced into 5 μm thick sections. In brief, slices were gradient dewaxed, washed in water, and heated up to 97 °C for 20 min in citric acid buffer (pH 6.0) for antigen retrieval. After cooling down, slices were incubated in 3% (w/v) hydrogen peroxide (H_2_O_2_) solution for 15 min to eliminate the effects of endogenous peroxidase and then blocked in 5% BSA solution for 30 min. Slices were infiltrated with anti-Ki-67 (ab16667, Abcam; 1:200) primary antibody at 4 °C overnight in a humid atmosphere. The next day, slices were washed and incubated with horseradish peroxidase (HRP)-conjugated secondary antibody for 1 h at room temperature. Color development reaction was performed for each slice by using freshly prepared 3,3-diaminobenzidiine tetrahydrochloride (DAB). Cell nuclei were counterstained with hematoxylin for 3 min. After staining, slices were sealed by neutral gum and images were captured by a microscope.

#### Flow cytometry analysis

To identify circulating tumor cells, we used a citrate-dextrose pretreated syringe to collect blood from mice by cardiac puncture when mice were scarified. After sedimentation of red blood cells with Ficoll (Sigma-Aldrich, USA), blood samples were washed for subsequent stainings. Cells were incubated in APC/Cy7-conjugated anti-human HLA-A, B, C antibodies (BioLegend, USA; 5 µl/10^6^ cells) for 30 min at 4 °C in the dark. To analyze MCT1 and MCT4 in primary and metastatic tumors, cells were stained with anti-MCT1 (20139-1-AP, Proteintech; 1:100) and anti-MCT4 (22787-1-AP, Proteintech; 1:100) primary antibodies for 30 min, followed by APC- (# A-10931, Invitrogen; 1:500) and FITC-conjugated (# F-2765, Invitrogen; 1:500) secondary antibodies for another 30 min, respectively. The corresponding isotype antibodies were used as negative controls. Stained cells were detected on a flow cytometer (BD Biosciences, San Jose, CA, USA). Data were analyzed by using FlowJo software (FlowJo, LLC., Ashland, OR, USA).

#### Statistical analysis

Before testing statistical significance of differences among various groups, we determined whether data were normally distributed and whether variance was homogeneous among groups. Normality was examined by Shapiro–Wilk test if 3 ≤ *n* < 20 (*n* means the number of samples) or D’Agostino Pearson Omnibus test if *n* ≥ 20. The homogeneity of variability was assessed by Brown-Forsythe test. When data were considered as normality (*p* ≥ 0.01) and equal variability (*p* ≥ 0.05), we performed one-way analysis of variance (ANOVA) followed by Holm-Sidak’s multiple comparisons adjustment. Otherwise, we assessed statistical significance of a difference by non-parametric Kruskal–Wallis test followed by Dunn’s multiple comparisons adjustment. To evaluate the statistical significance of differences for time-related data, we used two-way ANOVA followed by Tukey’s multiple comparison test. All of our statistical analyses were two-side and *p* < 0.05 was considered statistically significant. All statistical analyses in this study were performed by GraphPad Prism (Version 9.3.1) and all data were presented as mean ± standard deviation (SD).

## Results

### Inhibitions of MCT1 and/or MCT4 lead to a shift from glycolysis to mitochondrial respiration in tumor cells

**Fig. 1:**
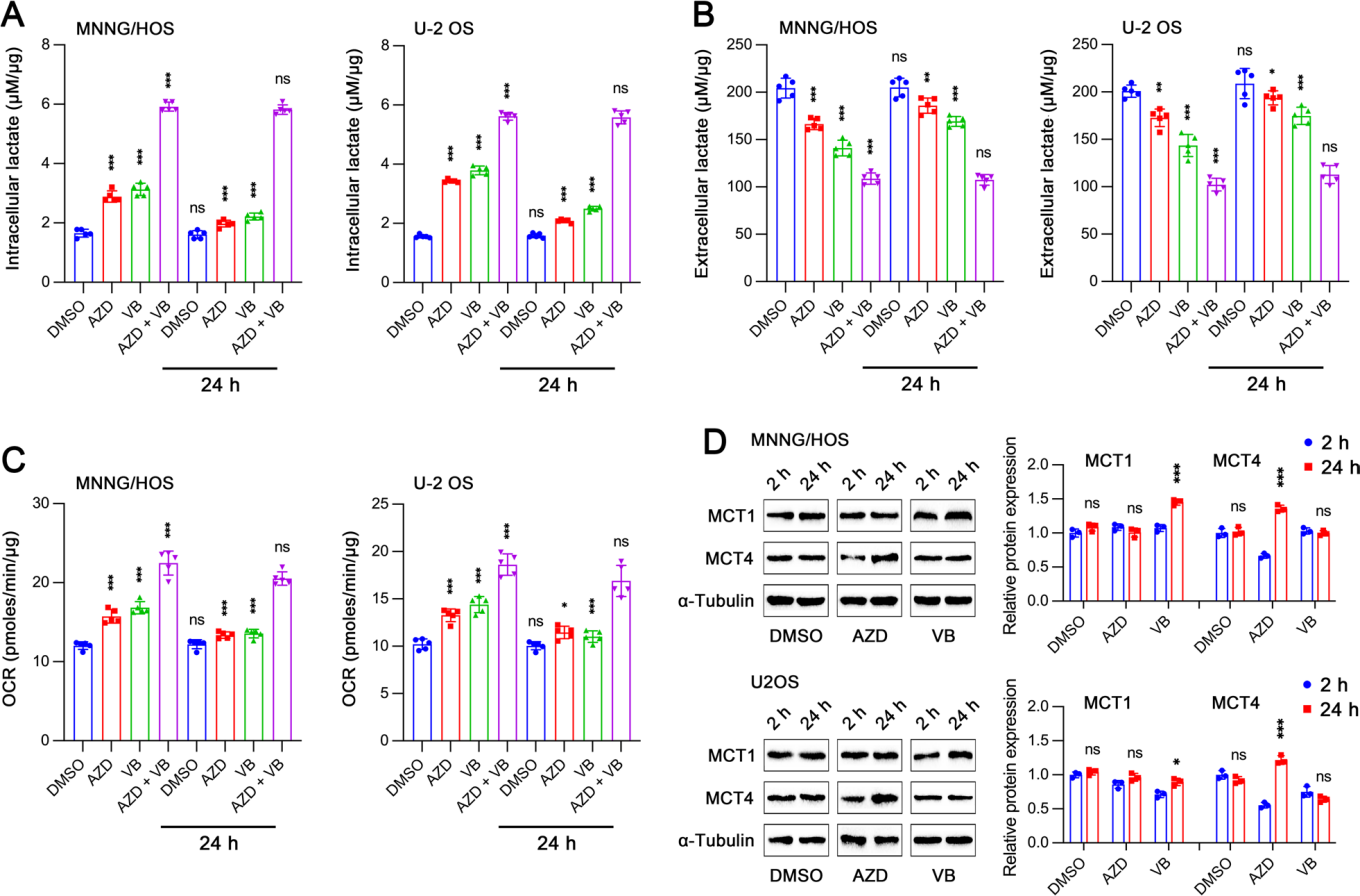
Effects of pharmacological inhibition of MCT1 and MCT4 on glycolysis and oxidative respiration. **A** Intracellular lactate levels in MNNG/HOS and U-2 OS cells treated with DMSO, 10 µM AZD3965 (AZD), 10 µM VB124 (VB) and AZD + VB for 2 h (*n* = 5). “24 h” means cells were pretreated with indicated reagents for 24 h and then intracellular lactate level was measured 2 h after replacement of culture medium. **B** Cells were treated as in **A** and extracellular lactate level was detected (*n* = 5). **C** Cells were treated as in **A** and basal oxygen consumption rate (OCR) was assessed (*n* = 5). **D** Protein expression of MCT1 and MCT4 by western blot analysis after treatment as in **A**. Data are shown as mean ± standard deviation (SD). In groups with treatment for 2 h, statistical differences are compared to DMSO group. In the groups with treatment for 24 h, statistical differences are compared to corresponding group with 2-h treatment. *p < 0.05, **p < 0.01, ***p < 0.001; ns, not significant

By using different concentrations of MCT and MCT4 inhibitors, we evaluated their dose–response effect on lactate transport (Additional file 1: Fig. S1A–H). Intracellular lactate was dramatically accumulated when either MCT1 or MCT4 was inhibited by 10 μM AZD3965 (AZD) or 10 μM VB124 (VB) for 2 h, respectively (Fig. 1A). AZD is a selective MCT1 inhibitor and has no activity against MCT4 [17]. Most recently, VB124 has been identified to be potent and selective for MCT4 over MCT1, which exhibits limited MCT1 inhibitory activity [18]. We observed a much stronger accumulation on intracellular lactate when both MCT1 and MCT4 were inhibited, suggesting a potential combined effect. In addition to such acute stimulation with MCT1/4 inhibitors, we also investigated the long-term effects of MCT1/4 inhibition on intracellular lactate level. Tumor cells were treated with AZD and/or VB for 24 h and then culture medium was replaced. After induction with the same treatment for another 2 h, intracellular lactate level was measured, which showed similar results to acute treatment for 2 h. Notably, long-term administration of AZD or VB alone had a weaker ability to increase intracellular lactate than transient administration, implying that long-term inhibition of MCT1 or MCT4 may lead to the transport of lactate out of cell through a compensatory mechanism. Extracellular lactate was also tested after treatment as in Fig. 1A and similar acute and long-term effects were observed (Fig. 1B). Either MCT1 or MCT4 inhibition could reduce basal oxygen consumption rate (OCR) both in MNNG/HOS and U-2 OS cells and combined inhibition of MCT1 and MCT4 displayed a prominent combination effect (Fig. 1C). We further explored whether AZD and VB had an effect on protein expressions of MCT1 and MCT4 (Fig. 1D). There was no obvious alteration of MCT1 and MCT4 expressions after treatment with AZD and VB for 2 h. Interestingly, long-term administration of AZD or VB resulted in the increase of MCT4 or MCT1 expression respectively, thus partially rescuing the effects induced by AZD or VB. Together, dual inhibition of MCT1 and MCT4 would lead to metabolic reprogramming by reducing glycolysis while facilitating aerobic respiration.

### Genetic deletions of MCT1 and/or MCT4 reduce glycolysis while enhances mitochondrial aerobic respiration

**Fig. 2:**
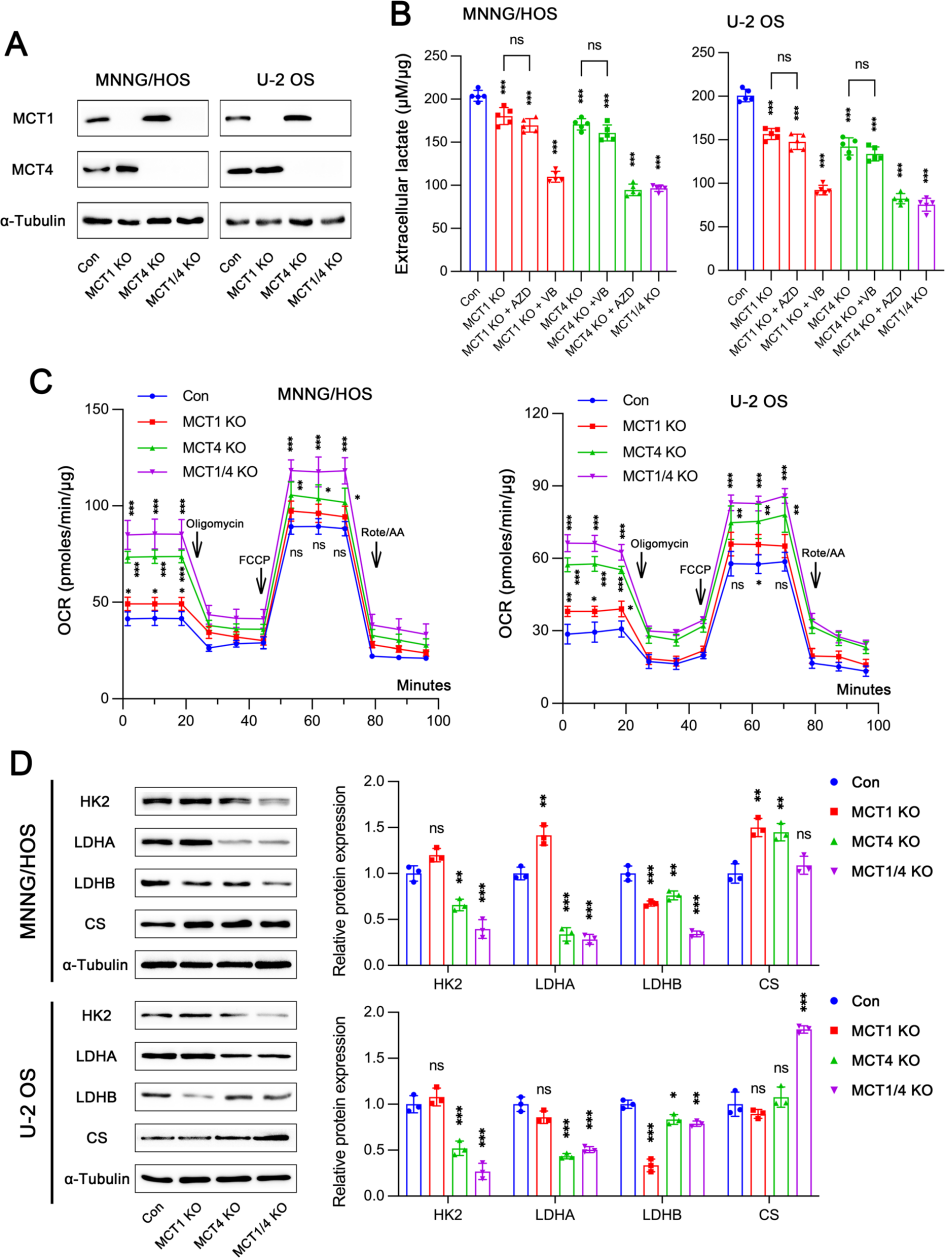
Effects of genetic knockout for MCT1 and MCT4 on glycolysis and oxidative respiration. **A** Protein expression of MCT1 and MCT4 in MNNG/HOS and U-2 OS cells after MCT1 and/or MCT4 knockout. **B** Extracellular lactate levels in MCT1 and/or MCT4 knockout (KO) cells treated with or without 10 µM AZD3965 (AZD) and 10 µM VB124 (VB) (*n* = 5). **C** Oxygen consumption rate (OCR) in MCT1 and/or MCT4 KO cells (*n* = 5). **D** Glucose metabolism-related protein expression levels in MCT1 and/or MCT4 KO cells. Data are shown as mean ± standard deviation (SD). Statistical differences are tested by comparison with control group, or as indicated. *p < 0.05, **p < 0.01, ***p < 0.001; ns, not significant

We constructed MCT1 and/or MCT4 knockout cell lines by using CRISP gene editing technology and validated them through western blot analysis (Fig. 2A). Knocking out either MCT1 or MCT4 resulted in a trend toward increased protein expression of the other, which was consistent with the consequences from long-term treatment of MCT inhibitors. Genetic deletion of either MCT1 or MCT4 was able to decrease extracellular lactate export, which could be dramatically amplified by simultaneous knockout of both MCT transporters (Fig. 2B). The specific activity of AZD3965 against MCT1 was verified again, which has been previously reported by various studies. It was noted that VB124 did not reduce extracellular lactate level in tumor cells after MCT4 deletion, confirming an on-target effect of VB124 on MCT4. This suggested that MCT1/4 deletion could markedly reduce glycolytic flux. Additionally, we also assessed mitochondrial oxidative phosphorylation capacity by measuring OCR. MCT1 and/or MCT4 knockout significantly enhanced oxygen-dependent glucose catabolism in MNNG/HOS and U-2 OS cells (Fig. 2C). Furthermore, glycolysis-related proteins including hexokinase 2 (HK2) and lactate dehydrogenase A (LDHA) were decreased by MCT4 deletion (Fig. 2D). Interestingly, MCT1 knockout could significantly inhibit LDHB expression. The expression of oxidative phosphorylation-related protein citrate synthase (CS) was increased to various degrees when MCT1 and/or MCT4 were knocked out. Notably, MCT1 KO significantly suppressed LDHB expression while MCT4 KO inhibited LDHA expression. Since MCT1 might be associated with uptake of extracellular lactate, which will be further transformed into pyruvate within tumor cell by LDHB. Consistently, OCR in MCT1 expressing cells (MCT4 KO) was higher than that in MCT4 expressing cells (MCT1 KO).

### Functional heterogeneity of MCT1 and MCT4 in glucose metabolism

**Fig. 3:**
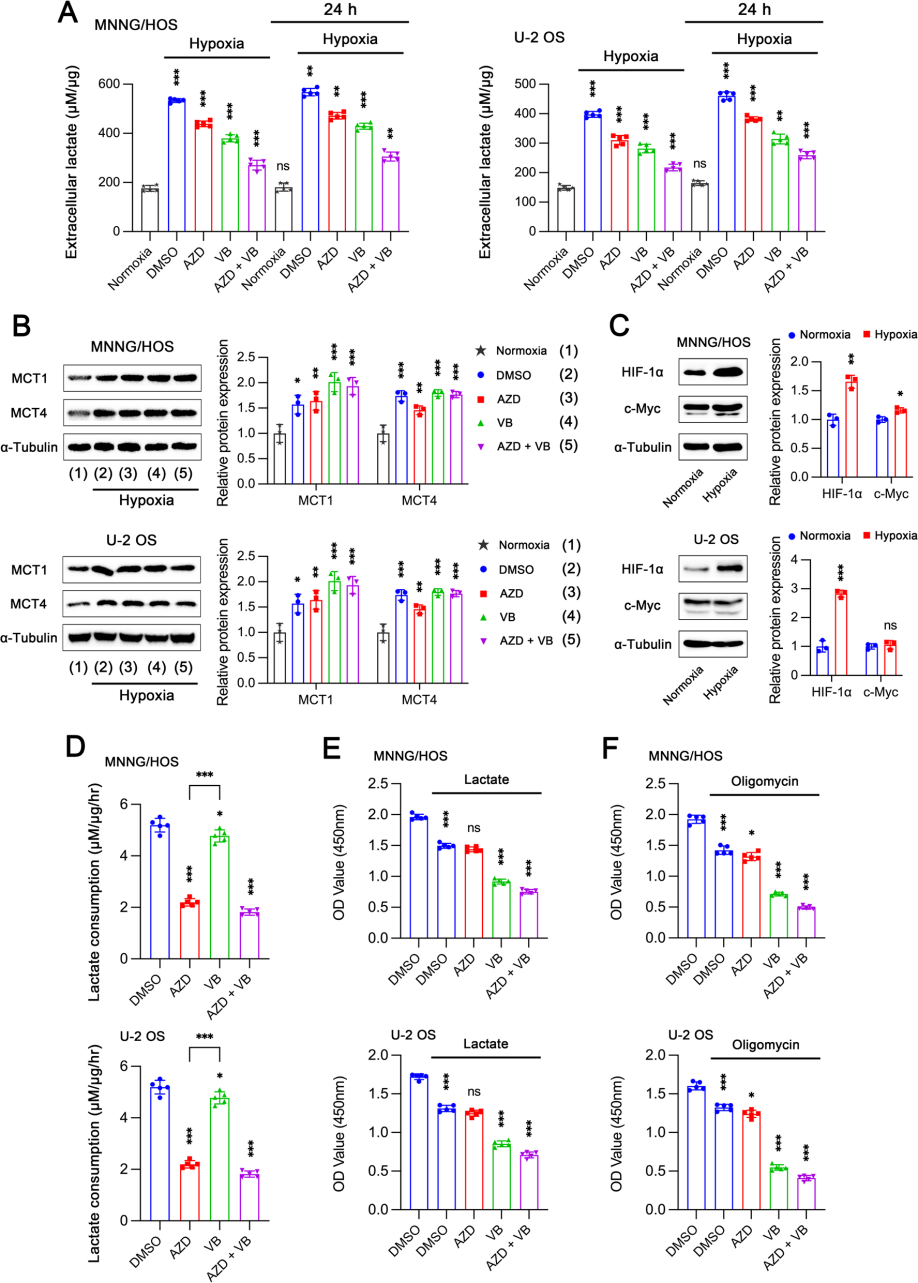
Effects of MCT1 and MCT4 on biological behaviors of tumor cells under different conditions including hypoxia, high concentration of extracellular lactate and inhibition of oxidative respiration. **A** Extracellular lactate levels in MNNG/HOS and U-2 OS cells treated with DMSO under normoxia, or with DMSO, 10 µM AZD3965 (AZD), 10 µM VB124 (VB) and AZD + VB under hypoxia for 2 h (*n* = 5). “24 h” means cells were pretreated as above for 24 h and then extracellular lactate level was measured 2 h after replacement of culture medium. **B** Protein expression of MCT1 and MCT4 in cells treated with DMSO under normoxia, or DMSO, AZD3965, VB124 and AZD + VB under hypoxia. **C** Protein expressions of HIF-1α and c-Myc in cells incubated under normoxia or hypoxia. **D** Lactate consumption in cells treated with DMSO, AZD3965, VB124 and AZD + VB for 24 h in low-glucose (500 mg/L) medium containing 20 mM lactate. **E** Cell proliferation analysis after treatment with DMSO, AZD3965, VB124 and AZD + VB in culture medium with or without 20 mM lactate for 3 days.** F** Cell proliferation assay in cells treated with DMSO, AZD3965, VB124 and AZD + VB, with or without 0.1 µM oligomycin (mitochondrial oxidative phosphorylation inhibitor) for 3 days. Data are shown as mean ± standard deviation (SD). In groups with treatment for 2 h, statistical differences are compared to normoxia group. In the groups with treatment for 24 h, statistical differences are obtained by comparing with corresponding group of 2-h treatment (**A**). In **D–F**, statistical differences are calculated by comparing to DMSO group or as indicated. *p < 0.05, **p < 0.01, ***p < 0.001; ns, not significant

Extracellular lactate was remarkably increased under hypoxia and MCT1 or MCT4 inhibition could reduce the extracellular lactate level, especially by dual inhibition (Fig. 3A). Besides, tumor cells released more lactate during 24 h of sustained hypoxia compared to 2 h of acute hypoxia, even in DMSO group. This suggested that prolonged hypoxia would trigger a systematically metabolic reprogramming to further enhance glycolysis. We found that both MCT1 and MCT4 expressions were significantly enhanced under hypoxia (Fig. 3B), which might be dependent on the increased HIF-1α and c-Myc (Fig. 3C). When cells were cultured in low-glucose (500 mg/L) medium containing 20 mM lactate, different concentrations of MCT and MCT4 inhibitors were used to assess the dose–response effect on extracellular lactate consumption (Additional file 1: Fig. S1I–L). AZD3965 could significantly inhibit extracellular lactate consumption, suggesting that MCT1 is the predominant transporter for lactate uptake (Fig. 3D). However, MCT4 rather than MCT1 endowed cells with survival advantage when facing high concentration of extracellular lactate (Fig. 3E), since VB124 could markedly reduce cell viability while AZD3965 could not. This result implied that MCT4 is able to transport lactate out of cell even under the condition of high concentration of extracellular lactate. In addition, MCT1 inhibition alone only acquired a modest effect on suppressing cell proliferation combined with the administration of oligomycin (oxidative phosphorylation inhibitor) (Fig. 3F). However, MCT4 inhibition could dramatically disrupt cell proliferation by combination with oligomycin. In summary, MCT1 can transport lactate in both directions, participating in both glycolysis and oxidative phosphorylation, while MCT4 is mainly responsible for glycolysis and transporting lactate out of cell.

### The effects of MCT1 and MCT4 on cell proliferation, migration and invasion

**Fig. 4:**
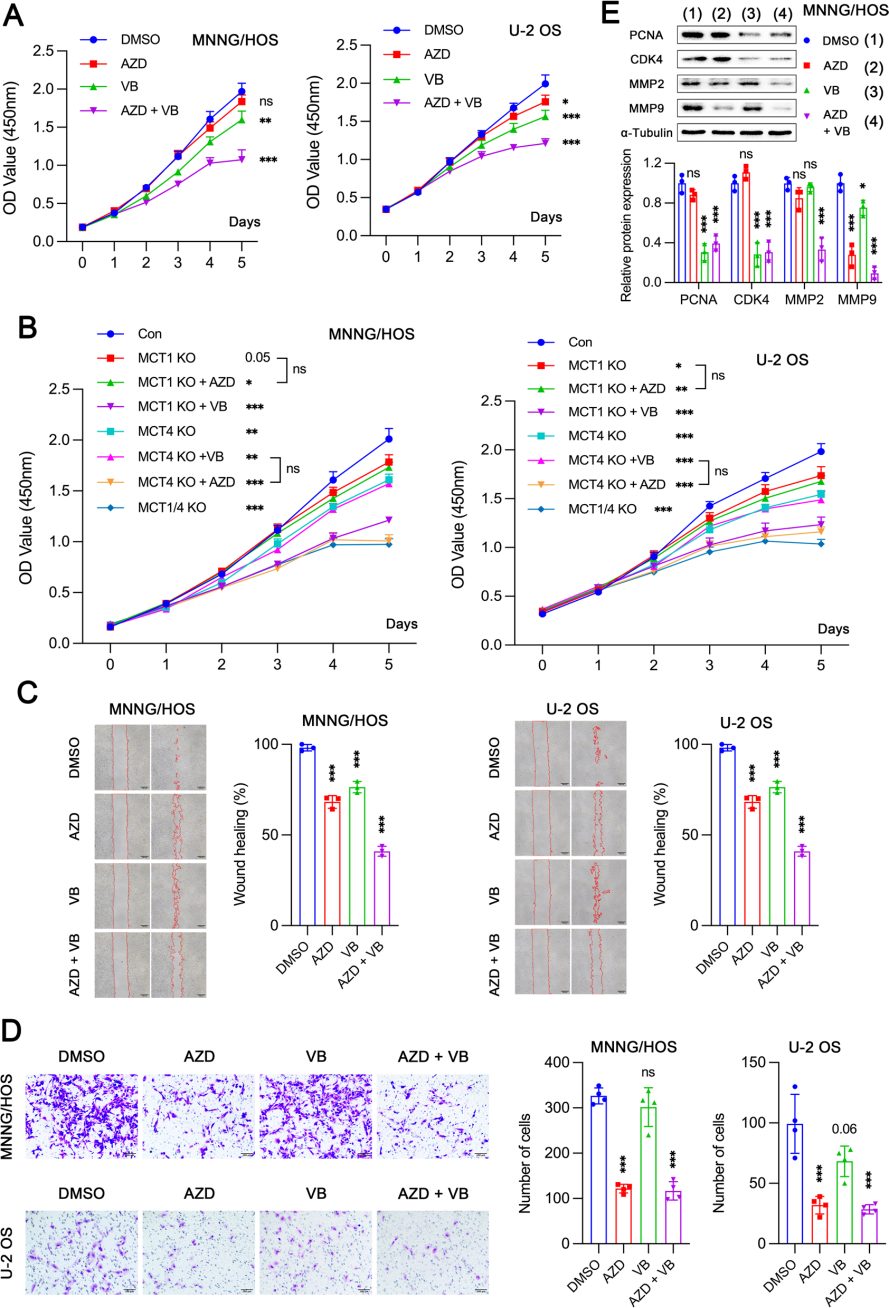
The role of MCT1 and MCT4 on tumor cell proliferation, migration and invasion. **A** Cell proliferation assay in MNNG/HOS and U-2 OS cells treated with 10 µM AZD3965 (AZD) and/or 10 µM VB124 (VB) (*n* = 5). **B** Cell proliferation assay in MCT1 and/or MCT4 knockout (KO) cells treated with or without AZD3965 and VB124 (*n* = 5). **C** The effect of MCT1 and/or MCT4 inhibitions on cell migration ability (*n* = 3). **D** Assessment of invasiveness in MNNG/HOS and U-2 OS cells after MCT1 and/or MCT4 inhibitions (*n* = 4). **E** Expression levels of proliferation- and metastasis-related proteins in MNNG/HOS cells treated with DMSO, AZD3965, VB124 and AZD + VB. Data are shown as mean ± standard deviation (SD). Statistical differences are tested by comparison with DMSO group, or as indicated. *p < 0.05, **p < 0.01, ***p < 0.001; ns, not significant

AZD3965 significantly inhibited cell proliferation in U-2 OS but not in MNNG/HOS, while VB124 could inhibit proliferation in both cell lines (Fig. 4A). The combination of AZD3965 and VB124 achieved a more pronounced suppression effect. Similar results were also obtained in MCT1 and/or MCT4 KO cells. We did not observe an inhibitory effect of AZD3965 and VB124 on proliferation in MCT1 and MCT4 KO cells respectively, further confirming their highly selective activity (Fig. 4B). Inhibition of both MCT1 and MCT4 could attenuate cell migration ability (Fig. 4C). Besides, it was noted that inhibitors of MCT1 instead of MCT4 markedly diminished invasiveness in both MNNG/HOS and U-2 OS cells (Fig. 4D). Consistently, AZD3965 treatment remarkably reduced the expression of metastatic proteins like matrix metallopeptidase 2 (MMP2) and MMP9, while VB124 administration caused a prominent reduction in the expression of proliferation related proteins including proliferating cell nuclear antigen (PCNA) and cyclin dependent kinase 4 (CDK4) in MNNG/HOS cells (Fig. 4E).

### MCT1 but not MCT4 inhibition impairs invasiveness of MNNG/HOS cells through inducing oxidative stress

**Fig. 5:**
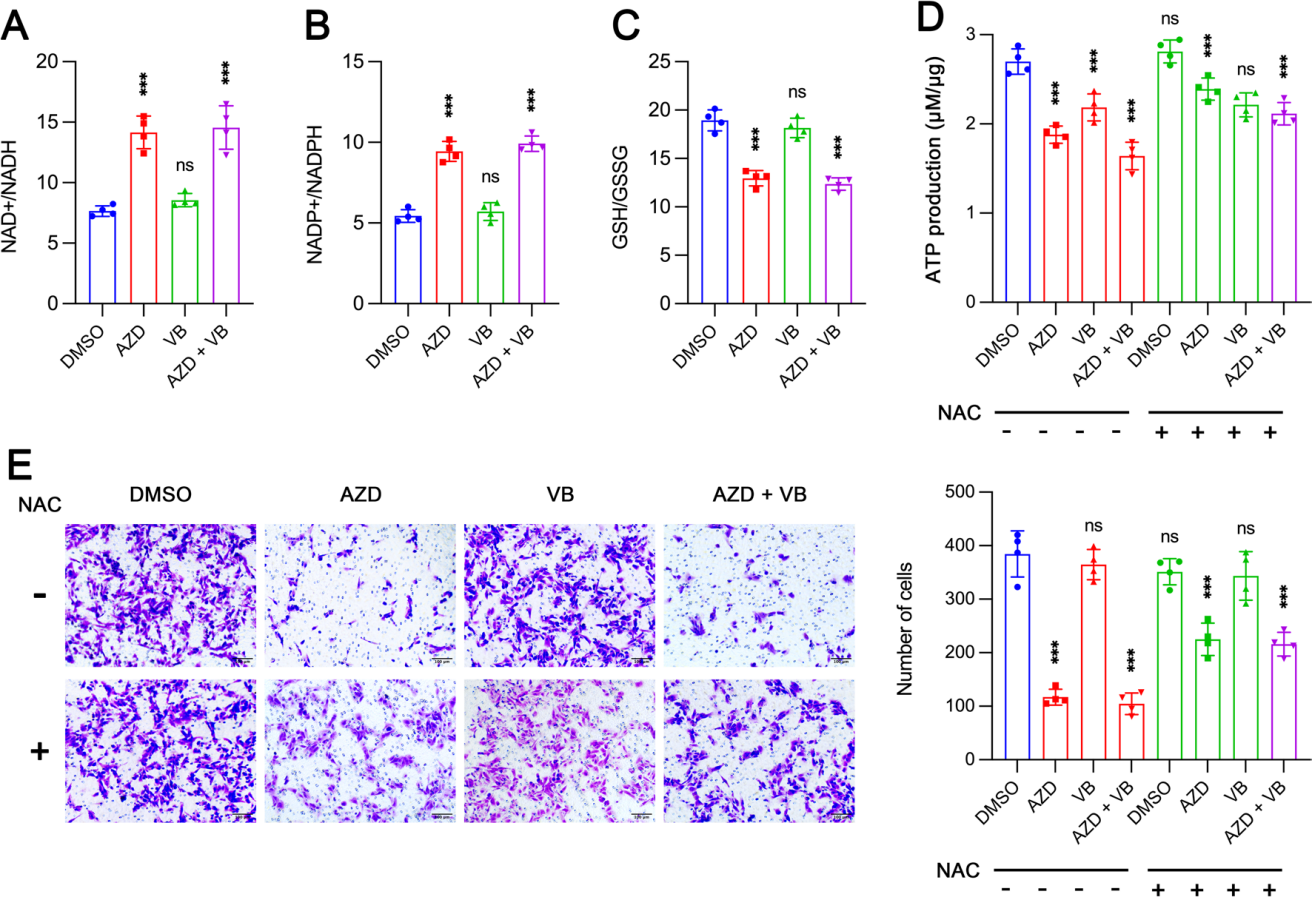
Inhibition of MCT1 but not MCT4 impairs metastatic capacity of MNNG/HOS cells by inducing oxidative stress in vitro. **A–C** NAD+/NADH (**A**), NADP+/NADPH (**B**) and GSH/GSSG (**C**) ratios in MNNG/HOS cells treated with DMSO, 10 µM AZD3965 (AZD), 10 µM VB124 (VB) and AZD + VB (*n* = 4). **D** The effects of MCT1 and/or MCT4 inhibitions on ATP production in cells treated with or without N-acetyl-cysteine (NAC) (*n* = 4). **E** Cell invasion assessment after MCT1 and/or MCT4 inhibitions and treatment with or without NAC (*n* = 4). Data are shown as mean ± standard deviation (SD). Statistical differences are obtained by comparison with DMSO group, or with corresponding group without NAC treatment. *p < 0.05, **p < 0.01, ***p < 0.001; ns, not significant

It was found that inhibition of MCT1, rather than MCT4, resulted in a significant increase in oxidative stress by measuring NAD + /NADH (Fig. 5A), NADP + /NADPH (Fig. 5B), and GSH/GSSG ratios (Fig. 5C). Both MCT1 and/or MCT4 inhibitions significantly reduced ATP production (Fig. 5D). Furthermore, administration of N-acetyl-cysteine (NAC), an antioxidant reagent, partially abolished the effect of MCT1 inhibition on ATP production, while having almost no activity on cells with MCT4 inhibition. Based on our previous results in this study, MCT1 was closely related to cell invasion ability. Thus, we examined whether NAC treatment could rescue the impaired cell invasiveness due to MCT1 inhibition. Tumor cells exhibited more aggressiveness after NAC application in AZD3965-treated group but not in VB124-treated group (Fig. 5E). Taken together, MCT1 inhibition could significantly damage the invasive capacity of OS cells via increasing oxidative stress.

### MCT1 inhibition modulates p65 distribution while MCT4 inhibition affects c-Myc expression

**Fig. 6:**
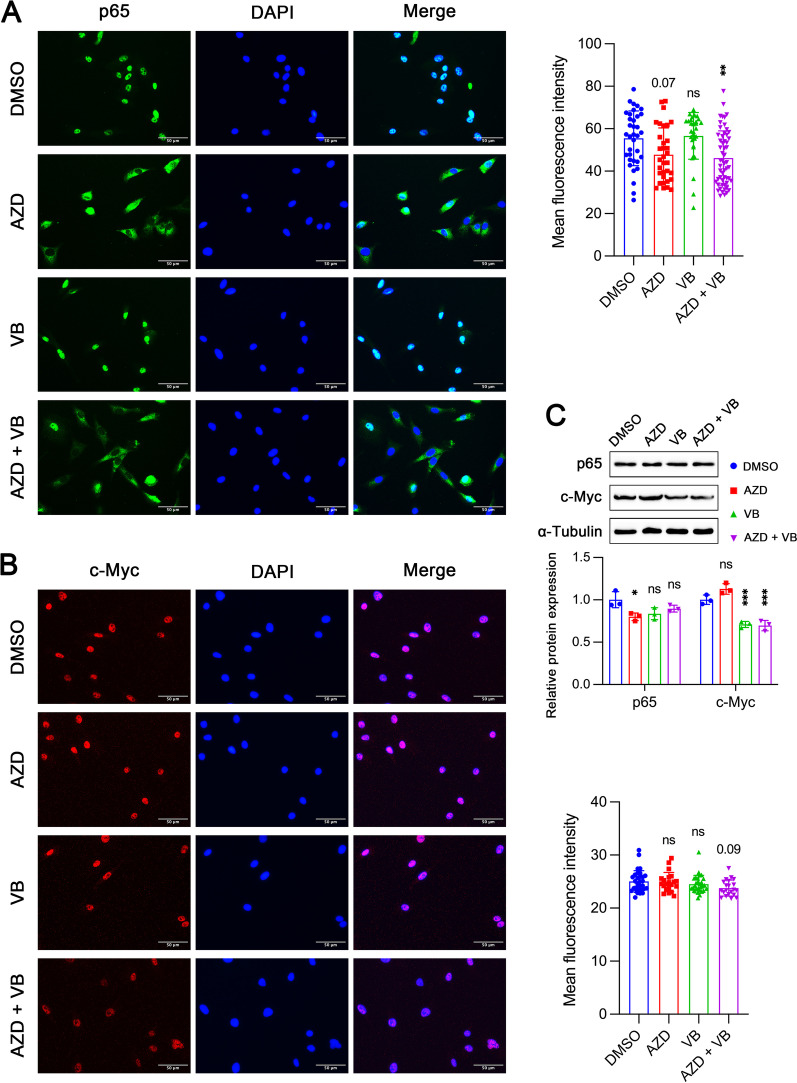
Effects of MCT1 and/or MCT4 inhibition on protein expression and localization of p65 and c-Myc in MNNG/HOS cells. **A** Representative immunofluorescence pictures and relative fluorescent intensity of p65 with treatment of DMSO, 10 µM AZD3965 (AZD), 10 µM VB124 (VB) and AZD + VB. **B** Representative immunofluorescence pictures and relative fluorescent intensity of c-Myc in cells treated with DMSO, AZD3965, VB124 and AZD + VB. **C** Protein expressions of p65 and c-Myc in cells treated with DMSO, AZD3965, VB124 and AZD + VB. Data are shown as mean ± standard deviation (SD). Statistical differences are obtained by comparison with DMSO group. *p < 0.05, **p < 0.01, ***p < 0.001; ns, not significant

We performed immunofluorescence staining for p65 in MNNG/HOS cells and found its translocation from nucleus to cytoplasm when MCT1 but not MCT4 was inhibited (Fig. 6A). In addition, neither MCT1 nor MCT4 inhibition seemed to have any effect on the distribution of c-Myc (Fig. 6B). The fluorescence intensity of both p65 and c-Myc was also not significantly altered after MCT1 and/or MCT4 inhibitions. However, protein expression of c-Myc was reduced when MCT4 was inhibited by VB124, while p65 expression was almost unchanged regardless of whether MCT1 or MCT4 inhibition (Fig. 6C).

### MCT4 deletion inhibits tumor growth while MCT1 deletion reduces metastasis in vivo

**Fig. 7:**
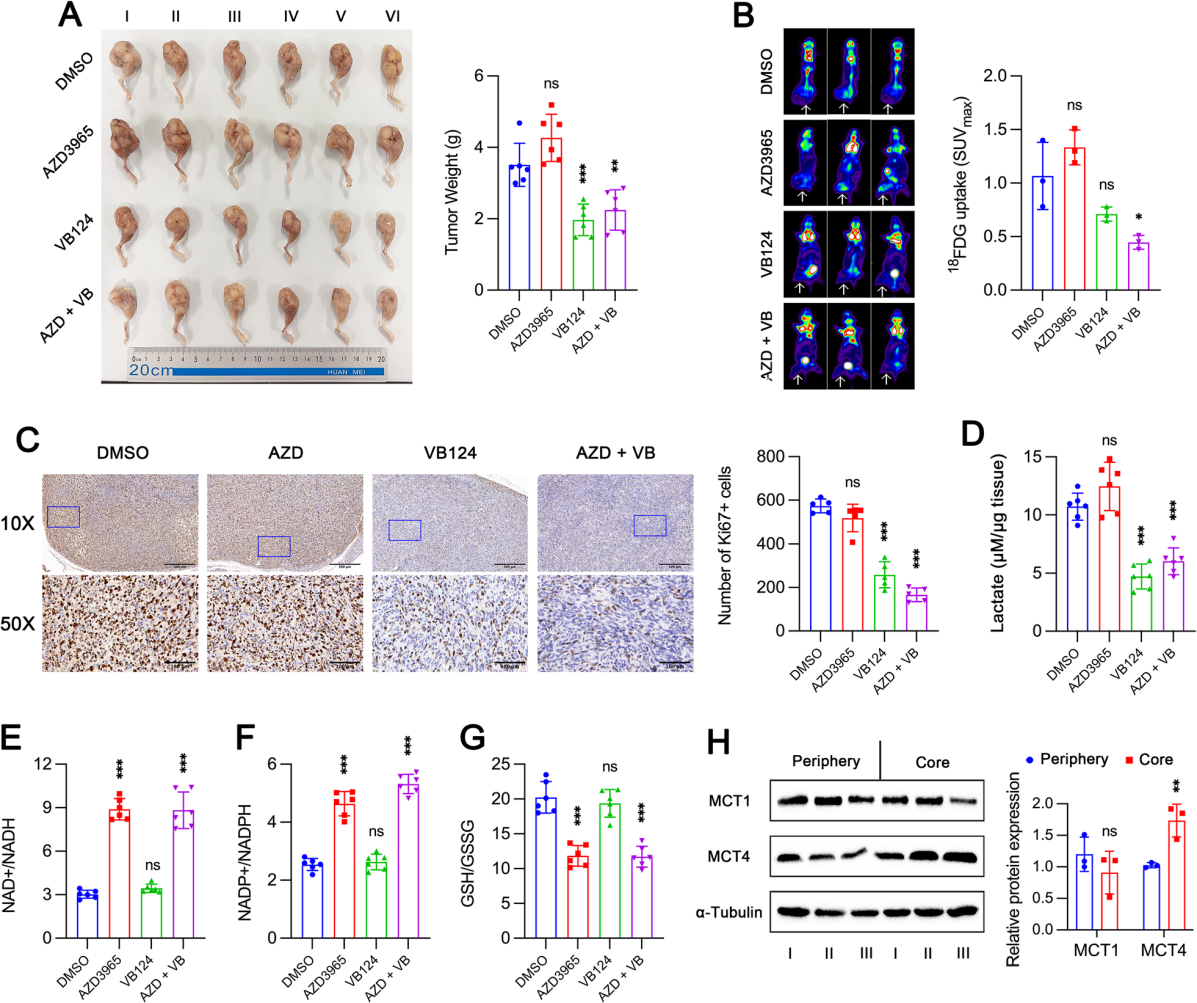
MCT4 deletion inhibits tumor growth while MCT1 deletion enhances oxidative stress in vivo. **A** MCT1 and/or MCT4 knockout (KO) MNNG/HOS cells were injected into bone marrow cavity of mouse femur (*n* = 6 for each group). Primary tumors were harvested and weighted after 4 weeks. **B** Evaluation of glucose uptake for mice from **A** (*n* = 3 for each group) by ^18^FDG PET/CT examination and tumor site indicated by arrows. **C** Representative pictures of immunohistochemistry staining of Ki67 and the number of Ki67-positive cells in primary tumor. **D** Evaluation of lactate levels within primary tumor tissue of mice from **A**. **E–G** NAD+/NADH (**E**), NADP+/NADPH (**F**) and GSH/GSSG (**G**) ratios analyzed in primary tumor tissue. **H** Protein expression levels of MCT1 and MCT4 in peripheral and core region of primary tumor tissue in mice from control group. Data are shown as mean ± standard deviation (SD). Statistical differences are tested by comparison with control group. *p < 0.05, **p < 0.01, ***p < 0.001; ns, not significant

We established an in-situ OS model in mouse femur by injecting control, MCT1 KO, MCT4 KO and MCT1/4 KO MNNG/HOS cells into the medullary cavity. Notably, MCT4 knockout dramatically inhibited tumor growth while MCT1 knockout did not alter tumor growth (Fig. 7A). MCT1 knockout led to a trend toward increased glucose uptake, while MCT4 knockout resulted in a trend toward reduced glucose uptake (Fig. 7B). Dual deletions of both MCT1 and MCT4 were potent to substantially decrease glucose uptake. These results suggested that tumor growth is more dependent on glycolysis and consumes more glucose when MCT1 is knocked out. The results of immunohistochemistry showed a significant decrease in the number of Ki67-positive cells in mice injected with MCT4 KO cells, whereas there was almost no difference in mice injected with MCT1 KO cells when compared to the control group (Fig. 7C). Similar results were also observed for the lactate level within tumor tissue (Fig. 7D). Consistent with in vitro experiments, deletion of MCT1 rather than MCT4 could induce oxidative stresses in vivo, as evidenced by increased NAD+/NADH (Fig. 7E) and NADP+/NADPH (Fig. 7F) ratios and decreased GSH/GSSG ratio (Fig. 7G). Importantly, the expression of MCT1 seemed to be higher in peripheral tumor tissue while MCT4 expression was significantly higher in core region of tumor tissue (Fig. 7H). Generally, it is more hypoxic within the core region of tumor tissue, where tumor growth relies more on MCT4-mediated glycolysis.

**Fig. 8:**
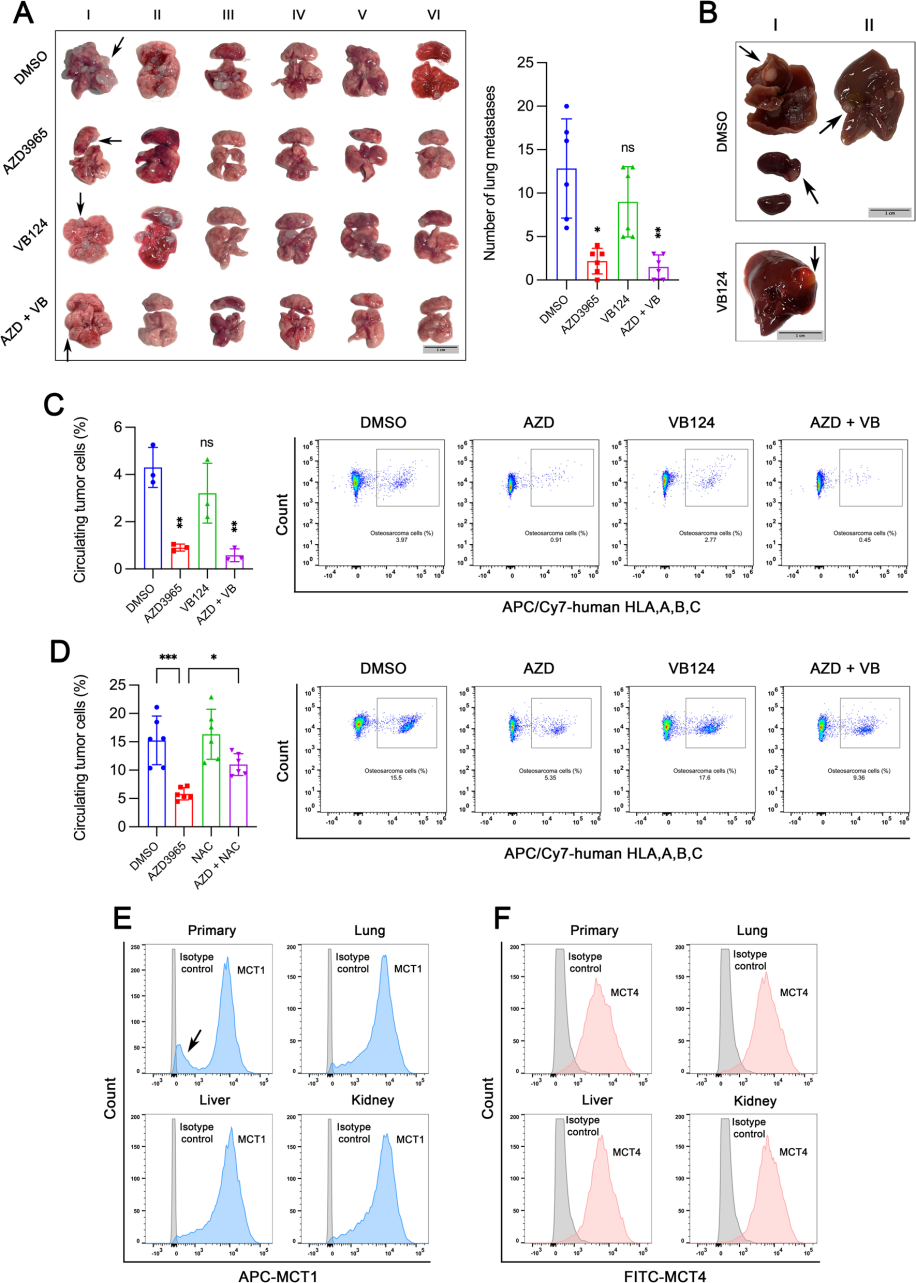
MCT1 deletion but not MCT4 significantly reduces circulating tumor cells and distant metastases. **A** Lung metastases in mice injected with control, MCT1 knockout (KO), MCT4 KO or MCT1/4 KO MNNG/HOS cells and the number of metastatic nodes was counted. Arrows indicated metastases. **B** Distant metastases that occurred beyond the lung including 3 mice in liver (2 from control group and 1 from MCT4 KO group) and 1 mouse in kidney from control group. Arrows indicated metastases. **C.** The frequency of circulating tumor cells in blood in mice from **A**. **D** Control or MCT1 KO cells were injected directly into the tail vein of mice followed by N-acetyl-cysteine (NAC) treatment or not and the frequency of circulating tumor cells was detected by flowcytometry after weeks. **E–F** Flow cytometry analysis of MCT1 (**E**) and MCT4 (**F**) expression patterns for primary tumor, and metastatic lung, liver and kidney tumor tissues in mice of control group from **A**. The arrow in **E** indicated a distinct cell subpopulation with very low expression of MCT1. Data are shown as mean ± standard deviation (SD). Statistical differences are tested by comparison with control group, or as indicated (**D**). *p < 0.05, **p < 0.01, ***p < 0.001; ns, not significant

Since lung is the most common site of distant metastasis in osteosarcoma, we assessed the effect of MCT1 and/or MCT4 deletion on lung metastasis. The number of lung metastatic lesions was significantly reduced when MCT1 KO cells were injected (Fig. 8A). Although we observed a trend toward decreased lung metastases in MCT4 KO group, there was no statistically significant difference compared to the control group. Interestingly, in addition to lung metastases, one mouse from control group developed liver and kidney metastases and another developed liver metastases, and one mouse injected with MCT4 KO cells suffered from liver metastases (Fig. 8B). Furthermore, we tested the frequency of circulating tumor cells in blood by flow cytometry analysis. MCT4 KO had little effect on circulating tumor cells, however, MCT1 KO substantially reduced the frequency of circulating tumor cells (Fig. 8C). This result suggested that MCT1 enables tumor cells to survive in the blood circulation during metastatic process. Then, we further investigated whether MCT1 conferred circulating tumor cell survival advantage through regulating oxidative stress. The decrease in frequency of circulating tumor cells induced by MCT1 deletion could be partially rescued by NAC treatment (Fig. 8D). In mice injected with control cells, flow cytometry was performed for primary and distant metastatic tumors to evaluate MCT1 and MCT4 expressions. Results revealed a shift in the expression profile of MCT1 (Fig. 8E) while MCT4 expression was almost the same (Fig. 8F) when comparing primary with metastatic tumors. In terms of MCT1 expression, there was a distinct subpopulation of cells with low MCT1 expression (MCT1^low^) in primary tumors (indicated by arrows in Fig. 8E), which could not be detected in metastatic tumor. This result implied that such MCT1^low^ cell population cannot survive or upregulate MCT1 expression due to various stresses encountered during metastasis. Therefore, osteosarcoma metastasis is largely dependent upon MCT1 and tumor growth primarily dependent on MCT4.

## Discussion

Transformed cells are intertwined with reprogrammed metabolism to support tumorigenesis and progression. Either as a cause or as a consequence, metabolic plasticity enables tumor growth and metastasis under specific microenvironment by adapting to biosynthetic and bioenergetic preferences. The intrinsic metabolism of cancer cell has attracted increasing attention [19], involving a variety of substances, such as glucose [20], lactate [12], lipid [21], amino acid [22] and so on. Although such metabolic remodeling confers a survival advantage to cancer cells, it also provides us with potential therapeutic strategies by targeting key metabolic nodes. In this study, we aimed to explore the role of MCT1 and MCT4 in metabolic remodeling and their effects on tumor growth and metastasis. MCTs, especially MCT1 and MCT4, play a critical role in tumor growth and progression, which has been reported in multiple types of cancers, including melanoma [16], lung cancer [23], breast cancer [24], hepatocellular carcinoma [25], osteosarcoma [15] and so on. Accordingly, much effort has been dedicated to the development of MCTs inhibitors with the aim of increasing the understanding of tumor metabolism and identifying promising anti-cancer agents. For MCT1, several potent and selective inhibitors have been developed [26]. For example, AZD3965 has been widely used to specifically inhibit MCT1 activity [17] and even initiated for phase I clinical trial (NCT01791595) [27]. A particular limitation of MCT1-specific inhibitors is that they might be ineffective due to MCT4 expression. Thus, dual inhibition of MCT1 and MCT4 can lead to a promising outcome in cancer therapy [13, 14]. Several dual inhibitors targeting MCT1 and MCT4 are already available. However, in order to study the individual function of MCT4 and the interaction between MCT1 and MCT4, it is still necessary to develop MCT4-selective inhibitors. The first selective MCT4 inhibitor (AZ93) was mentioned in 2015 but not disclosed [28]. Subsequently, several other MCT4-specific inhibitors were also reported briefly, which can be referred to a previous article [29]. Until 2021, two MCT4-specific inhibitors, VB124 [18] and MSC-4381 [29], were developed and well validated for their effectiveness in vitro and in vivo.

Here, we used AZD3965 and VB124 to inhibit MCT1 and MCT4 respectively and investigated their specific role in tumor growth and metastasis. We found that MCT1 is able to transport lactate bidirectionally across plasma membrane, into or out of cells. A subpopulation of tumor cells can take up extracellular lactate, generated from surrounding stromal and tumor cells or directly from circulation [12, 30]. The imported lactate will be converted to pyruvate, which subsequently enters mitochondrial for oxidative phosphorylation. Our results demonstrate that MCT1 participates in both glycolysis and oxidative respiration. MCT1 inhibition reduces the efflux of lactate produced by glycolysis and also reduces the uptake of extracellular lactate for further oxidative phosphorylation as well. In vitro, MCT1 inhibition substantially disrupts invasive capacity of tumor cells by inducing oxidative stress. Although MCT1 inhibition does not alter protein expression of p65, it induces translocation of p65 to cytoplasm. Additionally, MCT1 deletion significantly reduces lung metastases while has almost no effect on primary tumor growth. Mechanistically, MCT1 deficiency impairs the survival of circulating tumor cells during metastatic process. Moreover, the expression of MCT1 is found to be very low in a small unique group of cells at tumor primary site, whereas it would be upregulated in metastatic lesions. Consistently, the overall protein expression of MCT1 shows a higher level in metastatic tumor tissue, compared to primary tumor. In terms of MCT4, its inhibition significantly decreases extracellular lactate levels and suppresses cell proliferation. Remarkably, MCT4 plays a role in transporting lactic acid out of cell despite the high concentration of extracellular lactate, thus maintaining glycolysis. By combined treatment with oligomycin to block oxidative phosphorylation, MCT4 rather than MCT1 inhibition largely impairs cell proliferation, suggesting the casual role of MCT4 in glycolysis-dependent proliferation. MCT4 inhibition also reduces c-Myc expression, an essential transcriptional factor that regulates many glycolytic genes [31]. In xenografted mice, MCT4 deficiency substantially inhibits tumor growth with a tendency to reduced glucose uptake. And the lactate level in primary tumor is markedly decreased in mice transplanted with MCT4 KO cells.

Although lactate is not the only substrate of MCTs, MCTs-related lactate metabolism has become a research focus due to its high concentration in tumor tissue [32]. Once thought to be produced as a metabolic waste under hypoxia, we now know that lactate will be continuously formed even in the presence of sufficient oxygen. This phenomenon of aerobic glycolysis has been widely spread as Warburg effect in cancer [33]. Lactate can be utilized as a fuel for energy support and gluconeogenesis, as well as signaling molecule. Lactate fulfills these functions by being exchanged within and among cells and tissues, which is referred to as lactate shuttle [34]. Regarding intercellular lactate shuttle, driver cells produce lactate, which is then taken up and consumed by recipient cells. Here, our results suggest that tumor cells expressing MCT1 or MCT4 (glycolytic cells) produce lactate and those expressing MCT1 can take up and utilize lactate (oxidative cells). Consistent with previous studies, cells with high MCT4 expression mainly cluster in the central region of tumor tissue, where hypoxia is more pronounced, while cells with high MCT1 expression accumulate in the peripheral tumor. Moreover, hypoxia can significantly induce MCT4 expression [13, 35]. This position-dependent heterogeneity in MCTs expressions might contribute to lactate shuttle and thus support tumor progression. This metabolic symbiosis can be established between tumor cells with differential gene expression profiles by compartmental reorganization and functional complementarity, which is a potential mechanism underlying the resistance to antiangiogenic therapies [36–38]. In turn, lactate, as a metabolic signal, plays an important role in regulating various gene expressions such as MYC, HIF-1α and CDK4 as well as MCTs [39, 40]. Nevertheless, there are still many intriguing issues to be further addressed. For example, whether lactic acid produced by a cell will be re-uptaken by itself after being transported to extracellular region. If so, what is the meaning of this seemingly superfluous action. In addition, it is still controversial whether imported lactate is first converted to pyruvate in cytoplasm and then transported into the mitochondria, or directly transported into mitochondria for oxidation [41]. Moreover, which factors determine the direction of lactate transport by MCT1 and whether the direction of lactate transport by MCT1 is constant or variable for a single cell or single MCT1 molecule. In those tumor cells that generally express only MCT1 but not MCT4, whether MCT4 expression is induced in response to certain factors. In short, dual inhibition of MCT1 and MCT4 is able to efficiently break lactate shuttle in tumor tissues and thus inhibit tumor progression.

An equilibrated redox status ensures stable glucose metabolism, which in turn regulates redox maintenance at the organelle, cellular and tissue levels [42–44]. Lactate can influence cell redox, for instance by regulating reactive oxygen species (ROS) [45]. In addition, the redox state can also act as a force to drive the directional transfer of lactate. Importantly, cytosolic NAD+/NADH is closely related to cellular lactate/pyruvate ratio by MCT-mediated transport and LDH-mediated metabolic reactions. Glycolysis terminates in the conversion of pyruvate and NADH to lactate and NAD+. When MCT1 is present, extracellular lactate can be taken up into tumor cells and converted to pyruvate accompanied by the conversion of NAD+ to NADH [46]. Thus, MCT1 deletion dramatically increases NAD+/NADH ratios by blocking the conversion of NAD+ to NADH. Besides, MCT1 deficiency also increases NADP+/NADPH and GSSG/GSH ratios. Furthermore, when MCT1 is suppressed, administration of anti-oxidant NAC partially rescues ATP production and metastatic capacity both in vitro and vivo [47]. Therefore, MCT1 inhibition decreases metastasis at least partly by inducing oxidative stress.

There are some limitations in this study that need to be further elucidated. Although lactate is the most abundant substrate for MCT1/4 in human, the role of pyruvate in cancer cannot be ignored. More experiments are needed to explore the role of MCT-mediated pyruvate metabolism in OS progression and the interaction between pyruvate and lactate [18, 48]. The lactate level measured in our study in a static net level that cannot reflect the dynamic transport process. Since cancer cells will excrete as well as uptake lactate, we were unable to demonstrate the proportion of endogenous and exogenous lactate used by cancer cells. Notably, isotope labeling is an important method for studying the origin and fate of metabolites. By isotopically labeling glucose and lactate, we can trace their metabolic pathways and study the separate role of glycolysis and oxidative phosphorylation in OS growth and metastasis. Besides, we did not extract primary and metastatic OS cells from tumor tissue of mice and investigate their metabolic differences. Although we have suggested an association of MCT1 with p65 translocation while MCT4 with c-Myc expression, more research is necessary to further explore the potential mechanisms by which MCTs affect OS metabolism and metastasis. Our study lacks the evidence from clinical data. It will be more convincing to detect the expression of MCT1 and MCT4 in primary and metastatic tumor samples from OS patients, and to investigate the relationship between their expression and survival and tumor stage especially metastasis. Unfortunately, we failed to find any clinical evidence related to the expression of MCT1 and MCT4 in OS from public databases.

In our study, we investigated the functional differences and redundancies between MCT1 and MCT4 in glucose metabolism as well as in tumor growth and metastasis. MCT1 is implicated in both glycolysis and oxidative respiration due to its ability to transport lactic acid in both directions. MCT1 inhibition significantly reduces circulating tumor cells and thus impairs metastasis partially by increasing oxidative stress. MCT4 mainly contributes to glycolysis and is responsible for lactate export even in the case of high concentration of extracellular lactate. MCT4 expression is higher in the core of tumor tissue, where hypoxia-related glycolysis is more evident, compared to that in tumor peripheral regions. MCT4 deletion remarkably inhibits tumor growth with reduction of glucose uptake. Due to the functional complementarity and redundancy of MCT1 and MCT4, as well as metabolic symbiosis established based on the lactate shuttle, combined inhibition of MCT1 and MCT4 demonstrates the preclinical efficacy for osteosarcoma treatment. Further experiments are needed to study whether inhibition of MCT1 or MCT4 alone can achieve the same or better effect as combined inhibition in specific cases such as those without metastases.

## Supplementary Information

**Additional file 1: Figure S1**. Dose–response effects of AZD3965 and VB124 for MCT1 and MCT4 on lactate transport. **A** and **B** Intracellular lactate levels in MNNG/HOS cells treated with DMSO and AZD3965 (AZD) (**A**) or VB124 (VB) (**B**) at different concentrations. **C** and **D** Intracellular lactate levels in U-2 OS cells treated with DMSO and AZD (**C**) or VB (**D**) at different concentrations. **E** and **F** Extracellular lactate levels in MNNG/HOS cells treated with DMSO and AZD (**E**) or VB (**F**) at different concentrations. **G** and **H** Extracellular lactate levels in U-2 OS cells treated with DMSO and AZD (**G**) or VB (**H**) at different concentrations. **I** and **J** Lactate consumption levels in MNNG/HOS cells treated with DMSO and AZD (**I**) or VB (**J**) at different concentrations. Low-glucose (500 mg/L) medium containing 20 mM lactate was used. **K** and **L** Lactate consumption levels in U-2 OS cells treated with DMSO and AZD (**K**) or VB (**L**) at different concentrations. Low-glucose (500 mg/L) medium containing 20 mM lactate was used

## Data Availability

All data generated or analyzed during this study are available within the article or upon request from the author (GS, email: gaohongsheng@hust.edu.cn) with reasonable purpose.
