# Supplementary figures and images for: Functional heterogeneity of MCT1 and MCT4 in metabolic reprogramming affects osteosarcoma growth and metastasis
Source: J Orthop Surg Res. 2023 Feb 22;18:131. doi: 10.1186/s13018-023-03623-w (PMC9948327; doi:10.1186/s13018-023-03623-w)

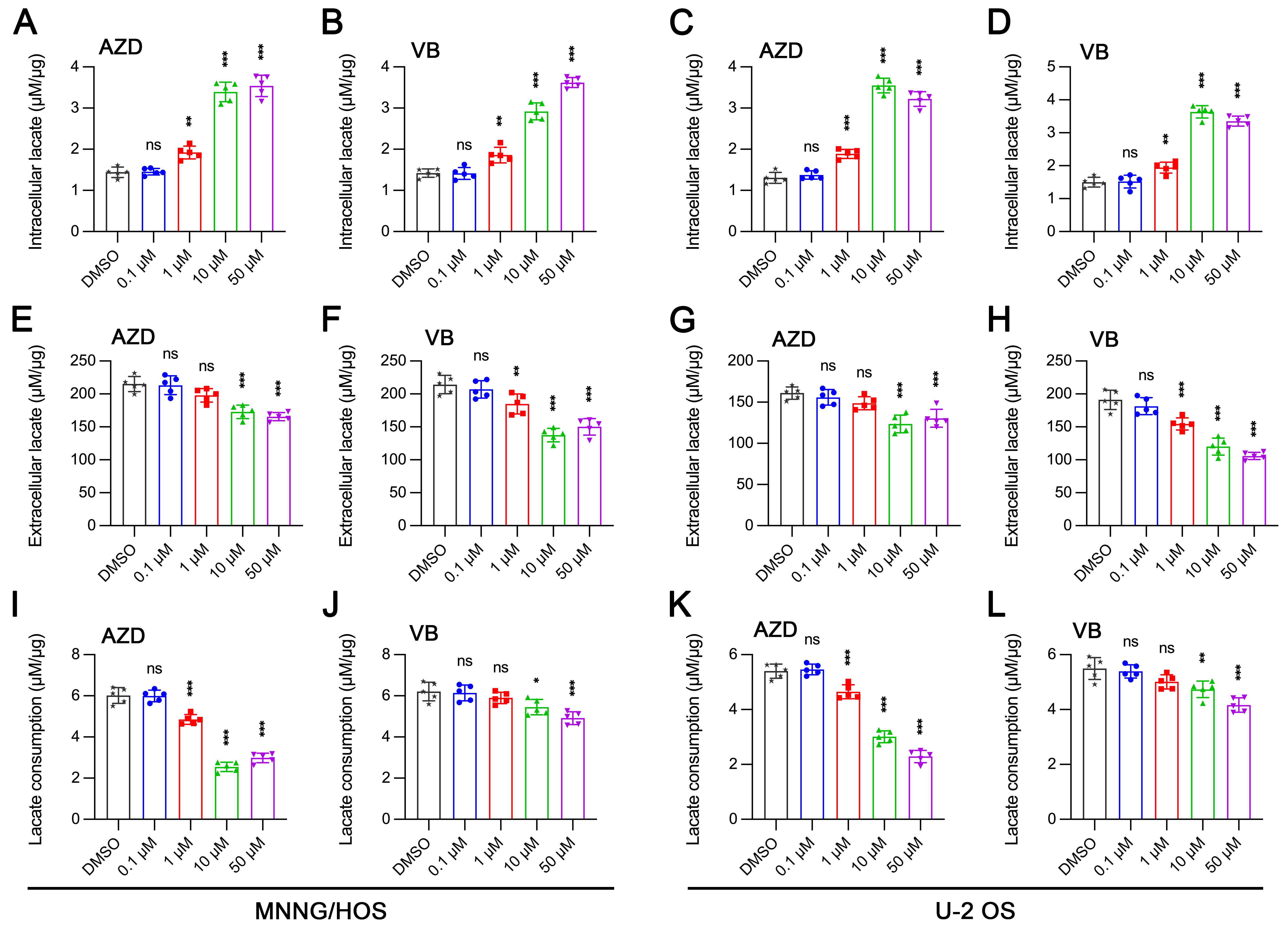

Supplement: Supplementary file 1 — Additional file 1: Figure S1. Dose–response effects of AZD3965 and VB124 for MCT1 and MCT4 on lactate transport. A and B Intracellular lactate levels in MNNG/HOS cells treated with DMSO and AZD3965 (AZD) (A) or VB124 (VB) (B) at different concentrations. C and D Intracellular lactate levels in U-2 OS cells treated with DMSO and AZD (C) or VB (D) at different concentrations. E and F Extracellular lactate levels in MNNG/HOS cells treated with DMSO and AZD (E) or VB (F) at different concentrations. G and H Extracellular lactate levels in U-2 OS cells treated with DMSO and AZD (G) or VB (H) at different concentrations. I and J Lactate consumption levels in MNNG/HOS cells treated with DMSO and AZD (I) or VB (J) at different concentrations. Low-glucose (500 mg/L) medium containing 20 mM lactate was used. K and L Lactate consumption levels in U-2 OS cells treated with DMSO and AZD (K) or VB (L) at different concentrations. Low-glucose (500 mg/L) medium containing 20 mM lactate was used [file 13018_2023_3623_MOESM1_ESM.tif]
